# Activities of Daily Living Ontology for Ubiquitous Systems: Development and Evaluation

**DOI:** 10.3390/s18072361

**Published:** 2018-07-20

**Authors:** Przemysław R. Woznowski, Emma L. Tonkin, Peter A. Flach

**Affiliations:** Faculty of Engineering, University of Bristol, Bristol, BS8 1UB, UK; p.r.woznowski@bristol.ac.uk (P.R.W.); Peter.Flach@bristol.ac.uk (P.A.F.)

**Keywords:** activities of daily living, ontology development, ontology validation, self-annotation, post-hoc annotation

## Abstract

Ubiquitous eHealth systems based on sensor technologies are seen as key enablers in the effort to reduce the financial impact of an ageing society. At the heart of such systems sit activity recognition algorithms, which need sensor data to reason over, and a ground truth of adequate quality used for training and validation purposes. The large set up costs of such research projects and their complexity limit rapid developments in this area. Therefore, information sharing and reuse, especially in the context of collected datasets, is key in overcoming these barriers. One approach which facilitates this process by reducing ambiguity is the use of ontologies. This article presents a hierarchical ontology for activities of daily living (ADL), together with two use cases of ground truth acquisition in which this ontology has been successfully utilised. Requirements placed on the ontology by ongoing work are discussed.

## 1. Introduction & Background

Healthcare needs have changed dramatically in recent times. An ageing population and the increase in chronic illnesses, such as diabetes, obesity, cardiovascular and neurological conditions, have influenced research, directing it towards Information Communication and Technology (ICT) solutions. These technologies make up ubiquitous systems, which quietly reside in the background and gather relevant, actionable information from various sources—mainly sensors. The idea of ‘calm computing’, first envisioned by Weiser [1] back in 1991, is now becoming a reality. Such systems are developed and used in the context of Ambient Intelligent (AmI) spaces, Ambient Assisted Living (AAL) and wearable healthcare systems, to name a few.

A large part of our lives is spent in the home—a proportion that increases as we grow older [2,3,4]—yet very little is known about our activities and behaviour in the home. Learning about the Activities of Daily Living (ADL) of people living in AAL spaces is key to answering many clinical questions, such as the cause and effect of various medical conditions or the effectiveness of various treatments and interventions. Efficient and accurate activity recognition (AR) algorithms [5] are needed in order to make sense of this data and provide useful/actionable information and services in the human activity monitoring context. Such an algorithm must produce machine-understandable data so that the data can be linked across many different domains. However, in order to achieve this goal, a ground truth is often required—that is, a dataset that can be used for training AR algorithms. Ground truth acquisition mechanisms aim to facilitate the development of AR algorithms by providing useful and interoperable activity labels, a task often achieved by manual *annotation* of some part or all of a dataset [6]. One way to facilitate this is to use formal information structures, such as ontologies, as underlying knowledge structures to support annotation. In this way the ambiguity of labels is reduced. The use of common models facilitates cross-discipline collaboration, knowledge exchange and reuse.

SPHERE (Sensor Platform for HEalth in a Residential Environment) [7] is one example of a data-intensive project in which the use of ontologies is beneficial. SPHERE is a large project consisting of a significant number of researchers, in which work is distributed across multiple work packages and universities. One group is responsible for the development of activity recognition algorithms, another group for developing mechanisms for acquiring ground truth and a third is responsible for the task of collecting data. All these groups must communicate and collaborate with each other as well as with other stakeholders. Furthermore, data collected throughout the course of the project is, where appropriate, retained for future researchers both within and outside the SPHERE project. This task requires a significant data curation and preservation investment; data curation requires a rigorous approach to description of the data and of the conditions under which it was created, including experimental hypotheses, design, methods and outcomes [8]. Standardisation in data encoding on every level, from the instance level to the metadata level, increases the reusability of datasets in general and is a foundational aspect of eScience [9].

Challenges and problems faced in the SPHERE project, especially in the context of standardisation in data encoding, reflect challenges that the AAL, AmI and pervasive systems communities face. The set up costs of such projects is very high, as is their complexity. Hence the most valuable and enduring legacies of projects of this kind are the captured and published datasets, which are not only the result of considerable financial investment but are also typically unique in their temporal and geographical coverage and in this sense may be regarded from a digital preservation perspective as non-replicable digital assets [10]. Despite this, however, we note that these datasets vary in quality, scale and potential for reuse within the scientific and engineering communities. In particular, datasets of this type are not always accompanied by a corresponding ground truth. The BoxLab project, introduced in the subsequent section of this article, lists datasets generated from instrumented living environments on their website [11]. A noticeable feature of these datasets is the lack of common models. Therefore, one conducting research in the area of AR would need, in the first instance, to adapt their code to fit each of the datasets. Secondly, they would need to map the set of concepts with which they work against those captured in each dataset. This leaves room for ambiguity and misinterpretation of the analysed data. Consequentially, the research community in this field would benefit from a well-designed, flexible and comprehensive ADL ontology to standardise such datasets. This work addresses this need by designing the presented SPHERE ADL ontology, promoting reuse and mapping to existing knowledge structures as far as possible, and ensuring that the newly designed ontology is iteratively validated and refined on the basis of data gleaned from real-world applications. This approach showcases our view of best practice in knowledge structure development as combining reuse of existing knowledge, standardisation and careful curation in response to real-world requirements and findings.

In this article, we begin by briefly identifying dominant themes in knowledge management mechanisms in smart home activity recognition. We then describe the ontology engineering approach used in the development of the SPHERE ADL ontology, introducing the ontology and its characteristics. We then demonstrate how case studies in the use of the SPHERE ADL ontology have been used to aggregate data about the ontology in use, thus allowing us to perform a series of data-and application-based evaluations of the ontology [12]—that is, through evaluation of the performance of the applications powered by the ontology, and of the data collected through those applications, strengths and weaknesses of the ontology itself are identified. This article extends preliminary work previously published by Woznowski et al. [13,14] by describing the development of the SPHERE ADL ontology, expressing its relationship to related knowledge structures and critically evaluating the ontology by reference to experience gained in its use in two case studies.

## 2. Literature Review

In this section, we first discuss knowledge representation artefacts designed for use with smart homes in particular, moving on to discuss classification schemes developed to support activity classification in Section 2.2. In Section 2.3 and Section 2.4, we discuss the process of ontology development and validation. Finally, we explore some of the sustainability issues facing knowledge representation artefacts beyond initial publication. Our intention is to identify resources presently available in the domain and to characterise them briefly in coverage and availability, to introduce the process of ontology development prior to a fuller discussion in Section 3 of this article, and to identify the pragmatic considerations underlying ontology selection and development.

### 2.1. Activity Recognition in Smart Homes

Several previous smart home projects exist, such as PRIMA [15], H-SAUDE [16] and Place Lab [17]. Of these, few have released openly available knowledge representation artefacts. H-SAUDE, a framework intended to support monitoring of hypertensive patients, explicitly discounts activity recognition as out of scope [16]. PRIMA describe the use of a ‘situation model’—an approach drawn from cognitive science, based on the work of Johnson-Laird [18]—to represent events and event sequencing. These situation models comprehend four general classes of data, including spatio-temporal information, entities (people, for example), properties of entities and relational information (people, kinship, time, place, causality, social links, etc.). The PRIMA framework, however, does not appear to have been published as an openly available knowledge schema. Place Lab developed an ontology to identify ‘objects, sensors and locations within the PlaceLab apartment’ [19]. The PlaceLab ontology was made available on the Web by the authors but, as is common with similar resources [20], is no longer available online. We identified that the resource remained available via the archive.org Wayback Machine [21]. We conducted an evaluation of the concepts covered in the PlaceLab ontology in order to establish the coverage of the ontology. From this, we found that the coverage of activities within the PlaceLab ontology is very limited and focuses on sensor output, such as estimation of motion magnitude, rather than on descriptive labelling at a higher level i.e., an estimate of motion magnitude may be encoded, but ‘climbing stairs’ or ‘washing dishes’ may not.

Riboni et al. [22] identify limited capacity for reasoning with temporal reasoning as a limiting factor in off-the-shelf Semantic Web technologies when used for activity recognition. Riboni and Bettini [23] later describe an OWL-DL (Web Ontology Language-Description Language) ontology comprehending various locations and activities. Chen et al. [24] describe a conceptual activity model based on ontology-based reasoning over granular information (time, activity, actor, location, resources, conditions, effects, goal, duration, environmental information).

Of the above knowledge resources, we identify several that are potentially of interest. However, we note that few of these knowledge representation artefacts are openly available for reuse. As d’Aquin and Noy [25] put it, ‘for ontologies to perform their role—facilitating interoperability between different systems and datasets—users must be able to find relevant ontologies quickly and easily’. Ontologies that have not been publicly released are difficult to find, access, evaluate and reuse. This seemingly trivial factor is a significant limitation in ontology reuse in this domain.

### 2.2. Taxonomies and Ontologies for ADL

One high-profile project engaged in the development and maintenance of a taxonomy covering activities of daily living is BoxLab—a project in the US funded by National Science Foundation, whose mission is to make home activity datasets a shared resource. On their website, one can currently find a number of hierarchical activity taxonomies available to download in XML format [11]; it is to be noted however that the site is expected to close and while the data owners have been contacted to identify a permanent home for this resource, it isn’t yet available at the time of writing. Definitions of all labels are also available. BoxLab captures a large number of classes. Within the BoxLab taxonomy, the *Activity* subsection has the following properties: *social context, activity* and *room location*. The *Abbreviated.Activity* subsection defines one extra property i.e., *posture*. The *Posture* subsection defined outside of the *Abbreviated.Activity* is actually labelled as *posture/ambulation* and has three subcategories: *posture, ambulation* and *transition*. How do these concepts connect together? Is *posture* conceptually different from *posture/ambulation*? Which one to use in which case? Are these concepts mutually exclusive? BoxLab also lists another two subsections called *Outside the home* and *Stereotypy*. While studying these resources, it is noticeable that there is no mention of the temporal factor. Obviously every activity starts at some point in time and finishes at another. Hence, even though BoxLab’s taxonomy came to be viewed as the best available candidate knowledge representation for use in the context of AAL/AmI activities within the SPHERE project, it was found that it lacks clarity and expressiveness in certain areas. Moreover, to the best of our knowledge no formal model of this ontology has been published in the literature, which limits the potential for standardisation or de facto widespread adoption.

Another example is the Compendium of Physical Activities (CPA)—a project supported by Arizona State University and National Cancer Institute in US. Ainsworth et al. [26] present their coding scheme for classifying physical activities by rate of energy expenditure. Since the initial publication, a number of updates were published. In the second update of this coding scheme, Ainsworth et al. [27] claim that, ‘Despite its known limitations, the Compendium has withstood the test of time to provide a valuable resource to code PA surveys or records and to provide examples of activities within a broad intensity range for use in PA counseling, research, and clinic settings’. On their website [28], the authors list 21 activity categories currently included in the CPA. The Home Activity category lists activities with corresponding codes. These activities are not organised hierarchically but rather make up a flat list that contains information which could be thought of as parameters of these activities. For example, ‘cleaning, sweeping carpet or floors, general’, ‘cleaning, sweeping, slow, light effort’ and ‘cleaning, sweeping, slow, moderate effort’ in CPA are three different activities. In fact, these can be thought of as one activity with different parameters, e.g., the effort level. At the same time the first activity in this list is very ambiguous as it ends with the word ‘general’. What is its relationship to the other two cleaning/sweeping activities? To summarise, the CPA classification coding scheme has not been expressed or published in the form of an ontology. Whilst this is not in itself a disqualifying factor, we found that it is capable of expressing fewer important attributes of activities than BoxLab’s taxonomies. It is, however, possible that a partial alignment between the two knowledge representation approaches could usefully be achieved in future.

### 2.3. Ontology Development and Validation

At its core, ontology development is a specialised knowledge engineering process. As such, a generic ontology development methodology is expected to include a number of core steps: to quote Uschold and Gruninger [29], one begins by identifying the purpose and scope of the development process, followed by the broad process of building the ontology. This step begins with capturing the elements required, key concepts and relationships contained within the defined scope. These elements may require some form of coding. Then the potential for reuse comes into play: if an existing ontology or ontologies are available that fulfil some part of the purpose and fall within scope, it may be preferable to reuse these in whole or in part rather than to develop a novel solution. Finally, one must judge the ontology’s fitness both as a knowledge structure and as a solution for a problem in its intended context of use—and in order to enable future reuse, the ontology must be documented.

This generic approach to ontology development is still essentially fit for purpose, although many enhancements have been proposed. For example, Noy et al. [30] propose the use of *competency questions* to evaluate a candidate ontology structure—questions that one must be able to answer using the knowledge structure that has been developed. In the same paper [30], the authors propose that a multi-stage approach be used, building one or more hierarchical taxonomies of concepts before proceeding to identify relevant properties. These together represent a terminological component—a conceptualisation of the concepts and relations involved in the domain, or, in computer science terms, a Tbox [31]. Once these are present, instructions such as domain and range may be identified.

The most interesting principle to emerge from Noy et al. [30]’s work is the statement that ‘ontology development is necessarily an iterative process’. The solution described by Uschold and Gruninger [29] is essentially a waterfall process, whilst Noy’s is iterative. In combination with another enhancement to the process of ontology development, the use of data (particularly free-text or semi-structured text) as a source from which to identify concepts for use in an ontology [32], we come to view the ontology as a perpetual work in progress.

To view each term present in an ontology within a Peircean triad implies the existence of three entities: there is the object signified (the object, location, activity, etc.); there is the sign (the term); there is the idea—the interpretation of that sign. As ontology engineers we would like the interpretation of the sign to be stable, for it to be consistent and for it to resemble our own expectations of interpretation. There is no guarantee that, even if we achieve this in the short term with a test user group, we can expect to achieve it over a longer period of time without active maintenance; the user’s interpretation is not under our control. Observing patterns of use of an ontology through examination of the outcomes of case studies is expected to support the process of identifying shortcomings and potentially beneficial enhancements or changes under at least some conditions; we will explore this expectation in this article.

### 2.4. Beyond Development—Sustainability and Reuse

Whilst significant effort has clearly gone into development of ontologies in smart homes and into related areas such as context-awareness, it is notable that relatively few of these resources remain available in the long term. Indeed, a study undertaken in 2012 [20] explored the longer-term survival of ontologies identified in a literature review of knowledge structures in the domain of context-awareness. Twelve were identified by literature review. Of these, it was found that only four of these knowledge structures were available online at the time of the study, whilst copies of a further two could be found on archive.org. The rest either had never been made available or were no longer available online, even in digitally preserved copies of websites.

d’Aquin and Noy [25] identify a similar effect, not only identifying ontology discovery as a problem but also remarking that ‘ontology libraries’, systems that have been designed to support ontology discovery across the Web, vary significantly in scope and editorial policy and are likely eventually to fall out of use. Thus, whilst there are many ontologies described in the literature, we have found that few of these have progressed to publication as information artefacts. Relatively few are available for reuse today, either because they were never published, or as a consequence of ‘link rot’ [33]. Fewer still are taken up by the community to the extent that they become de facto standards, or are brought forward towards eventual formal standardisation by committees such as ISO, W3C or DCMI. Despite prior standards efforts such as the W3C Semantic Web in UbiComp Special Interest Group [34], we are not aware of any standard, de facto or otherwise, currently extant in this domain.

We see the processes described in this article as relating not solely to the development and validation of an ontology structure, but also to the ontology’s longer-term relevance, validity, persistence and sustainability. Although publication is the first step to encourage community uptake and reuse, it is not sufficient. The standardisation process requires an often lengthy incubation period of engagement with standards committees and with the communities for whom the work is of potential interest.

Following initial publication, we have taken several steps towards eventual standardisation. We have sought to map our work against an existing knowledge structure, demonstrating compatibility with at least one existing work in the field, a process that will be discussed in Section 3.1. We have built a practice-based approach to learning from each context in which the ontology is used, providing us with an evidence-based means to gauge relevance and validity in the field, an approach that will be discussed in Section 4. In formally publishing a version of the ontology at an early stage and reporting on the work at domain-relevant workshops [35], we have sought to gather feedback and encourage reuse by others working in the domain, with some success (see Discussion).

The remainder of this article introduces the SPHERE ontology of activities of daily living in detail. It also demonstrates the use of this ontology in two use cases. The first of these use cases consists of a post-hoc observer annotation of scripted experiments and the second is an unscripted self-annotation in free living. These studies are reflected upon from a machine learning perspective. We then provide a brief introduction to our current work, which explores the use of the SPHERE ADL ontology in the context of clinical studies. This article then concludes with the Discussion and Conclusion sections.

## 3. Development of the Ontology for Activities of Daily Living

As Gruber [36] puts it, an ontology is ‘a treaty—a social agreement’. It is a specialised representation artefact, designed to usefully mediate between an artificial network of labelled concepts and the perceptions and classifications of the people who work with it. The development of the SPHERE ADL ontology is the end result of a knowledge management process that took place within SPHERE, and which is discussed in detail in Section 3 and Section 4 of this article. SPHERE did not begin with the expectation that an ontology would be required; rather, the working group began with an investigation of the project’s data management and data annotation requirements. A second, parallel strand of activity involved researching available knowledge structures such as ontologies already available in this space, with the expectation that the project would, in accordance with best practice in the area, reuse existing domain knowledge where possible.

These requirements emerged when designing ground truth annotation mechanisms and mostly came from the machine learning group, for whom it was important to capture all the contextual and spatio-temporal information in order to validate their multi-residency AR algorithms. During the ground truth data collection phase of developing platforms for healthcare such as SPHERE, it is important to acquire information such as: activity, social context, physical state, interaction with objects, spatio-temporal data and physiological context.

The SPHERE ontology for activities of daily living has been developed and constructed to list and categorise activities occurring in the home environment. The initial purpose of the ontology included requirements that implied collection of a broader set of contextual information, such as the room in which events occur within the home and elements of physiological state. Additionally, it was expected that data collection would take place at several levels of granularity over time, according to the purpose of each specific research study. Hence it became necessary for the ontology to provide both fine-grained (i.e., individual, granular elements of activities) and broad-brush levels of description (for example, ‘cooking’).

The initial dictionary of ADLs was compiled during project meetings between researchers from the SPHERE project and clinicians and is reported on by Woznowski et al. [13]. The result of this collaborative effort has been extended for completeness (by which is meant adequacy of coverage sufficient to respond to all defined competency questions [30]) mainly with activities found in the Compendium of Physical Activities (see Section 2.2).

None of the reviewed taxonomies and ontologies fully satisfied the requirements of the SPHERE project: in particular, no single ontology could be found that covered all of the relevant concepts and relations—in the sense later described in Section 4.1, the relevance and completeness of these resources was typically low, resulting in limited expressiveness in the intended domain of use. However, we found that BoxLab’s published taxonomy represented a partial match to our requirements. Therefore, rather than crafting a wholly original ontology, we elected to make use of a hybrid approach. Whilst we created a new ontology, we elected to map our work closely with BoxLab’s where possible, reusing and extending concepts from the existing taxonomy. In such a way, we hope to facilitate data reuse between projects making use of either the BoxLab taxonomy or the SPHERE ADL ontology.

The final stage involved merging it with BoxLab’s taxonomy (see Section 2.2). This process of ontology alignment was considered to be beneficial, since compliance with existing models ensures interoperability and applicability of collected datasets beyond the project. Of the available knowledge representation structures, we found that Boxlab’s hierarchical taxonomies were the nearest fit to SPHERE in terms of both functional requirements (coverage, extent, etc.) and non-functional requirements. This evaluation was made by comparing our initial dictionary of ADLs and associated expressions, in the form of a hierarchical taxonomy, with each of the candidate knowledge structures. In particular, these resources had been published online in a computer-readable structured format that fostered ease of reference. Non-functional requirements that we were unable to wholly satisfy were: alignment with formally published standards and specifications, due to the dearth of relevant formal standards; full structural alignment, due to the variation in structures used in this domain; syntactic harmonization with best practice in the domain, as this is so variable. The alignment between SPHERE’s ADL ontology and BoxLab is depicted graphically later in the article (see Section 4.3.1).

### 3.1. Characteristics of the Ontology for Activities of Daily Living

Structurally speaking, BoxLab’s model could be viewed as a series of two-tiered taxonomies of labelled concepts, separated into general categories and specific subcategories. The SPHERE ADL ontology originates in a similar hierarchy of concepts, developed following Noy’s methodology [30] with reference to BoxLab’s work, and tested using competency questions developed within SPHERE.

**Figure 1:**
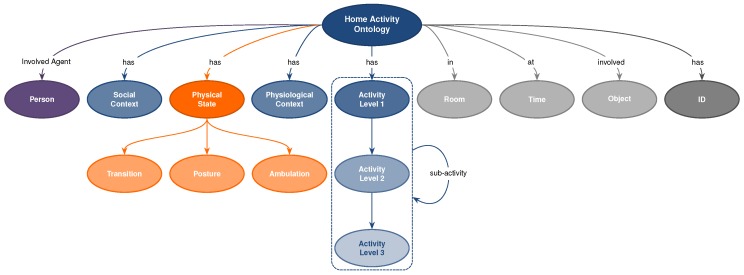
High-level view of ADL ontology.

To construct the ontology, we made use of the OBO-Edit (Open Biomedical Ontologies) ontology editing and construction tool. This tool is built for and primarily used by biologists and is quite widely used in the biomedical domain [37] and an informal assessment of the proposed domain of use indicated that the standard was more widely accepted than OWL in the healthcare domain at the time. This decision coloured the immediate future of the ontology, as the OBO tool is principally intended for the construction of controlled vocabularies. The hierarchy found in Figure 1 represents a high-level view of the full ontology.

In line with BoxLab’s model, the root concept of the taxonomic hierarchy links to the following child concepts: *activity, physical state* (posture/ambulation in BoxLab’s model), *social context* and *room location*. Furthermore, as represented by Figure 1, the ontology has been extended with additional concepts and properties, namely *physiological context, (at) time, (involve) object, (involvedAgent) person, ID,* and *sub-activity* with a self referencing relation. Thus, each activity that occurs at a certain point in time can be identifiably and labelled by some unique ID. Moreover, any given activity can involve physical object(s) and people. Any given activity might be made up of a number of *sub-activities*. Physiological context/signals are also important due to the fact that healthcare is the primary application of this ontology, alongside health and social care related tasks that involve monitoring people living independently in the safety of their own homes; physiological context is of particular importance in many use cases of this kind. Overall, AAL technologies have to ensure the user’s safety to an acceptable level of reliability and monitoring physiological signs is a significant component technology to support solutions of this kind. The present-day market of wearable sensors and smartphones reflects this interest, as products increasingly offer the ability to measure and monitor physiological information such as, for example, heart rate monitoring features.

**Figure 2:**
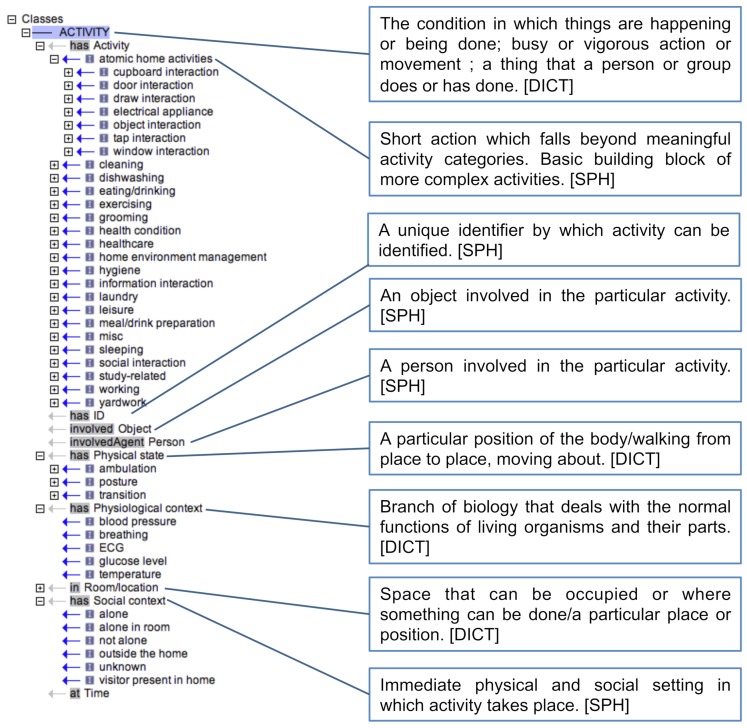
High-level overview of the entire ontology. The origin of each definition is indicated in square brackets. DICT is taken from a dictionary and SPH is taken from in-house documentation.

Figure 2 depicts the structure and class definitions of the SPHERE ontology for ADL, the latest version of which, in the OBO [38] format, is available from the data.bris repository (refer to the Supplementary Materials note).

### 3.2. Activity Hierarchy

In the SPHERE ADL ontology, ADLs are organised hierarchically. *Activity* has 20 sub-classes out of which 15 are present, albeit some names may differ slightly to better reflect classes listed in the subclasses below in the BoxLab taxonomy.

Activities often involve interactions with one or more objects. These interactions/activities have been reflected in the ontology in the *Atomic home activities* class and its subclasses. These capture the low-level activities or simple actions (evidencing sub-activities) which form the basic building blocks for other activities. One can use these labels to identify short actions for use in AR algorithms. With the increasing sophistication of wearable technology and sensing, research into identifying these types of activities will become more prominent. In the current version of the ontology, *Atomic home activities* has the following subclasses: *door interaction, window interaction, object interaction, tap interaction, cupboard interaction, draw interaction*, and *electrical appliance interaction*, each with a further level of subclasses (omitted for brevity).

The *Health condition* class is essential to describe activities and behaviours in the context of a person’s health. By training algorithms for AmI or AAL applications and associating level of participation in activities and incidence of symptoms with a person’s well-being, early warning signs that someone is unwell or in need of assistance or medical treatment could be predicted. This is especially important given the health challenges currently facing society and their inherent socioeconomic impact. This category currently includes: *coughing, fall, fever/infection, shaking* and *sweating*.

*Social interaction* is comprised of: *receive visitors, social media, talking* (with subclasses), and *video calling* activities. Finally, *working* is further divided into *intellectual* and *physical work*. Every subclass of *activity* (listed in Table 1) has a *misc* member to enable annotation of knowledge which the ontology does not explicitly capture. *Misc*, in Table 2, is for activity labels which do not fit into any of the existing classes. For example *smoking tobacco* has been added to this subclass as a result of clinicians’ feedback.

### 3.3. Ambulation, Postures, and Transitions

The structure of *Physical state* directly reflects BoxLab’s ontology *posture/ambulation* category, yet has been extended with additional entities. It has three subclasses, namely *ambulation, posture*, and *transition*. Table 3 provides the hierarchy of *Physical state* classes listing the number of subclasses and examples for each. Since activities do not always describe a person’s posture (with some exceptions, e.g., *running* where the posture is inherent in the activity), it is important to capture this information separately.

### 3.4. Contextual Information

*Room/location, social context and physiological context* make up contextual information. For any activity it is beneficial to know the context in which it occurred. Some activities are closely associated with a particular location (bathing activity in bathroom location) where some can occur anywhere inside or outside the home environment. From the healthcare perspective it is also important to capture social context as people’s behaviour can be affected by presence of other individuals. Finally, physiological context such as blood pressure or glucose level influence our well-being and behaviour. Information captured without context is of limited value as it does not fully reflect reality. The classes and examples of the three contextual information categories described above are provided in Table 4. In addition, activities contain reference to *(involved) object* and *(involvedAgent) person* properties, which capture object(s) and people involved in a particular activity. Since some activities can be made up of shorter (in duration) activities the *sub-activity* relation was introduced. For completeness, a (has) ID attribute was introduced to differentiate between activities. All these properties and relations are captured in Figure 1.

## 4. The Role of Case Studies in Validation and Revision of the SPHERE ADL Ontology

Since the initial release of the SPHERE ADL Ontology, it has been used in a number of operational contexts both within and external to the SPHERE project. In this section, we discuss several ongoing use cases, reviewing the SPHERE ADL ontology against a set of design characteristics drawn from the literature. For this purpose, we make use of information drawn from two completed pieces of work in which the ADL ontology was used in practice to support the collection of annotation datasets: a post-hoc video annotation task and a contemporaneous self-annotation task in a naturalistic environment [39].

### 4.1. Method

Table 5 provides a number of design characteristics that are potentially of relevance for an ontology. These are primarily taken from a large review of ontology evaluation strategies presented by Degbelo [40], who presents design evaluation criteria for use during the ontology design phase. We have adopted an iterative development approach throughout the lifecycle of the SPHERE ADL ontology up to the present, according to the recommendations laid down by Noy et al. [30], and hence cyclically re-evaluate in response to new data.

Following Kuziemsky and Lau [41], we note that ontology design is challenging in the biomedical domain for many reasons. These include misunderstanding of user needs, misunderstanding of context and poorly articulated user needs. Methodologically, therefore, a knowledge construct such as an ontology or taxonomy is expected to benefit from an iterative engineering approach, as these issues can be rectified at a later stage.

We combine two approaches to the review of our ontology. Firstly, we review lessons drawn from the practical use of the ontology in the following two case studies. This domain-specific evaluation explores the interaction between the ontology construct and the user conceptualisation of the domain, helping us to identify any mismatches between the two. Secondly, we compare the characteristics of the ontology against a basket of desirable characteristics drawn from best practice and guidance in the domain—see Table 5.

The collection of data from real-world uses of the ontology permits an understanding of where and how the ontology is used. Collection of additional information using other modalities—for example, interviews with users of information systems and evaluation of parallel knowledge management infrastructure used in the same environment—provides the opportunity to evaluate not only where an ontology is used, but also where it is not used.

We begin with the most straightforward of these, the use of the ontology within an annotation tool used by a post-hoc observer working from scripted, recorded data, in this case video data [42]. The second case is the use of the ontology within an annotation tool [14] intended to support unscripted annotation in free living within a smart home environment, in this case a home in which the SPHERE system is deployed.

### 4.2. Post-Hoc (Retrospective) Observer Annotation of Scripted Experiments

As discussed earlier, the development of ambient assisted living systems frequently mandates collection of ground truth annotations. These are primarily used to support the training and testing of models able to provide reliable predictions of aspects of human activity.

Whilst the ultimate goal is to enable reliable prediction in free-living contexts i.e., identifying unscripted activities in as naturalistic a dataset as possible, the practicalities of system development mean that this is ordinarily a multi-stage process. We began with a series of scripted activities, noting that each of the activities selected could be represented using the SPHERE ADL ontology—had this proven not to be the case, it would imply either that the task was out of scope for the ontology, which we did not believe to be the case, or that the ontology required further refinement.

Video data was collected during each scripted experiment, using a head-mounted video camera. This data was then annotated. Initially, the ANVIL tool was used for this purpose [43]. However, the team subsequently adopted the ELAN annotation tool, developed by MPI Nijmegen [44] for the purpose of creating complex annotations on video/audio resources [45]. ELAN, initially designed to permit annotation with arbitrary vocabulary, has been extended for ontology-based annotation [43]; this functionality can also be effectively simulated by mandating the use of a controlled vocabulary for a given ‘tier’ (annotation layer).

ELAN is based around the concept of annotation of a timeline, and therefore, of events with a non-negligible duration. There is a possibility that imposition of constraints can avoid invalid descriptions, such as an individual being reported to appear in two rooms simultaneously, which are reported to occur in similar place-based annotation datasets [19]. At present, this is left to the interface. The potential for temporal representation and reasoning within the ontology itself remains, although there is limited support within OBO for automated reasoning and validation (discussed below).

#### Concept Usage

**Figure 3:**
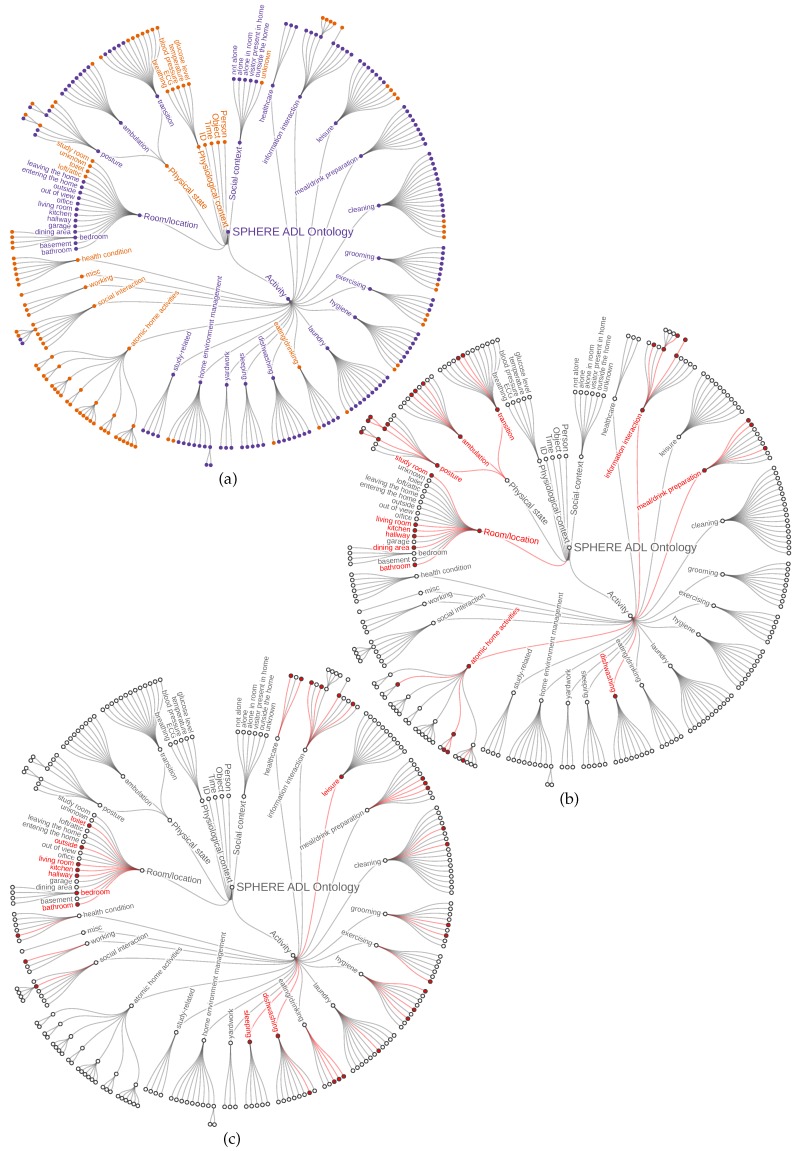
SPHERE ontology terms mapped to the Boxlab taxonomy and to two case studies. (**a**) SPHERE ontology (orange) alignment with BoxLab (purple). (**b**) SPHERE ADL terms used in post-hoc video annotation. (**c**) SPHERE ADL terms used in self-annotation condition (terms used for this purpose are highlighted in red).

A limited number of concepts are used in this task. ELAN is most straightforwardly used for the annotation of a limited number of data types at a time—for example, one timeline might contain verbs of motion and action and a second might contain nouns signifying location. Figure 3b displays a typical subsetting of the SPHERE ADL Ontology for this purpose. Only the subset of vocabulary depicted in bold font is offered for annotators to use. Typically, it is provided as a small number of flat keyword lists, ordered and presented to suit usability requirements and facilitate the task; annotators are limited to the use of this controlled vocabulary.

It is worth noting that the labels given to annotators are typically shortened and simplified by comparison to the concept label present in the ontology itself, although they are used in a semantically equivalent manner. This is consistent with the common practice of hiding ontologies ‘under the hood’ in user interface design [46]. The requirement for annotation is to provide a semantically equivalent cue that imposes minimal cognitive load and is straightforwardly understandable, whereas the labels used within the SPHERE ADL ontology itself are selected for accessibility and consistency of interpretation rather than for brevity. Since a mapping exists between the key terms and the ontology, it is reasonably straightforward to formalise the encoding at a later time. For example, this step might be taken as part of a strategy of standardising data and annotations in order to support data publication.

### 4.3. Unscripted Self-Annotation in Free Living

Annotation in free-living is a significant component in the validation of potential solutions for AAL, despite the attendant complexities. Several factors complicate self-directed annotation, such as the complexity of any available interface and the inherently problematical nature of any means of documenting an activity that inherently requires that activity to be put on hold—this is discussed in [39].

For the purposes of SPHERE, an application was developed for the Android operating system that allows for flexible, time-based annotation using terms selected from the SPHERE ontology of ADLs. As a first step, we collected a series of functional and non-functional requirements (functionality, input and output, usability, security, privacy and conditions of use). Reviewing available applications, we could not find an off-the-shelf solution that fulfilled these requirements. Therefore, we elected to develop an application ‘in-house’ on the basis of the Android application framework, using Android Studio. This was provided to study participants in the SPHERE project who elected to stay in the initial SPHERE pilot install home, a two-storey building near the university with a well-tested and effective sensor network home installation. This application supported a variety of interaction modes including voice input (via a speech-to-text service), menu navigation and RFID/NFC ‘tap’ functionality.

An initial review of the outcomes from this study is available [14]; a detailed review of study outcomes is currently under review. A relevant subset of these findings are presented here, since these represent a data source for validation of the ADL ontology in a real-world context of use.

#### 4.3.1. Concept Usage

In this case, participants were able to select from several annotation methods, including unstructured text and voice data submission, structured selection from a taxonomy ordered hierarchically by the location in which activities primarily took place, and NFC tagging. In order to present this data effectively, the data has been normalised against the SPHERE ADL ontology—that is, each term has been linked to the node to which it corresponds. This normalisation process has provided a useful opportunity to identify concepts that may not be included in the dataset, and these will be discussed later in this article as part of the validation section.

This dataset is revealing of users’ own perceptions and preferences in annotation. It is notable (see Figure 3c) that participants choose to annotate tasks at a different level to that used in annotation of scripted activities. Notably, participants often selected activities that mapped directly with BoxLab’s taxonomies (see comparison with Figure 3a).

## 5. Validation Outcomes

### 5.1. Relevance

The SPHERE ADL ontology fulfils the requirements of a manual post-hoc annotation process such as the case described above. It also fulfils the majority of the requirements for user-contributed annotation. Viewed solely through the lens of the two case studies above, there are a large number of unused terms, such as granular descriptions of cupboard use, information about social context, etc.

Both case studies make significant use of location. One limitation occurs here: homes vary significantly in their layout, and participants’ preferred nomenclature also varies, often significantly, both between participants and over time. It is not unusual for room function to change, particularly when something changes in a participant’s lifestyle or medical circumstances. For example, a participant suffering from illness or injury may find it preferable to relocate to a floor that reduces their need to use the stairs, changing their bedroom to a different location. A change of working habits might provoke the redecoration of a room into a study. Additionally, under some circumstances the name that a room is given might depend on circumstance, as with a kitchen/diner—a participant who is currently cooking might refer to it as the ‘kitchen’, whilst later it might be referenced as the ‘dining-room’.

Semantic variations of this nature are internally consistent only if adequate models of user behaviour and of the use of the property are kept over time. This echoes the earlier view of user interfaces as independent entities: an ontology, far from being the backbone of all interactions, is only one of several representations.

### 5.2. Consistency

A detailed review of the data returned in our second case study, reported and analysed by Tonkin et al. [39], demonstrates that term use is not consistent in the case of self-reported data. In particular, we note that terms that might reasonably be expected to occur symmetrically, such as ‘making a drink’ and ‘drinking a drink’, in fact appear to be used interchangeably. This is particularly common with shorter schemas, i.e., ‘making a meal’ and ‘eating a meal’ are relatively symmetrical in occurrence. We conclude that shorter activities are more likely to be annotated at only one point in the process, and that the preferred subtask for annotation varies between individuals.

### 5.3. Completeness

Missing values identified through the case studies above include:

- descriptions of certain activities, such as, in the self-annotation case, packing and unpacking suitcases—since participants in the self-annotation experiment were staying for several days in SPHERE’s property, this is an activity that many participants could be expected to undertake.
- transitions between rooms, which are not currently encodable.
- descriptions of certain parts of the home, notably stairs; this absence became evident during data analysis, but possibly resulted from the activity-centric origins of the ontology.

### 5.4. Structural Clarity

An automated validation of the SPHERE ADL ontology was completed. We established that the ontology could not be a valid tree by inspecting node and edge count, since node count must be equal to edge count + 1 if the graph has a valid tree structure.

Using the obonet package [51], we then loaded the ontology into Python’s Networkx package and used Networkx’s *find_cycle* method to identify the cyclic subgraphs present within the structure. These occur primarily in cases in which multiple inheritance is employed. A child class with two parents (i.e., child is-a-kind-of parent1 and child is-a-kind-of parent2) becomes represented as a cyclic subgraph when loaded from an OBO representation. As a data structure, the cyclic subgraph causes various practical problems with graph manipulation, as ontologies are ordinarily represented in tree structures and algorithms are typically written with the expectation of a tree structure. Software intended for use with ontology structures is not always ‘loop-safe’. Consequentially, the use of multiple inheritance breaks many tools. It is possible to explicitly express multiple inheritance in OWL; however, it is often recommended that this be avoided as far as possible since many software packages do not allow for the practice.

**Figure 4:**
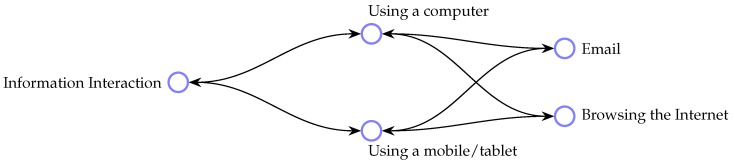
Two cyclic subgraphs.

We ultimately identified two classes that inherited from multiple parents: term 183 (Email) and term 191 (Browsing the Internet), both of which can be achieved using multiple classes of device. A hierarchical relationship of this kind may be read according to the following rule: if class *A* is a superclass of *B*, then every *B* must be a kind of *A* [30]. Therefore, the structure shown in Figure 4 represents an ambiguity of representation.

‘Email’ and ‘browsing the Internet’ are information interaction activities which can be completed using a computer or a mobile phone. Hence, one possible means of addressing this issue is to encode this information using an alternative method, such as the use of a ‘using’ relationship and more explicitly expressing the intended connection between the concepts ‘computer’, ‘mobile’, ‘email’ and ‘browsing the Internet’.

### 5.5. Expressiveness

In ontology design, particularly in the theoretical underpinnings of OWL, the open-world assumption (partial knowledge of the world) is made—i.e., what we do not know, we cannot guess at one way or another. It is not valid to make the assumption that, for example, any individual whose social context is not specifically defined must be ‘alone’. This echoes our experience of the ways in which the SPHERE ADL ontology is used. Pragmatically, most uses of the SPHERE ADL ontology are not exhaustive—rather, they are partial annotations, making use of a task-relevant subset of the ontology. Despite the benefits of rich annotation, even a post-hoc annotator is unlikely to annotate exhaustively for reasons of time, practicality and scarcity of resources. Participant-driven annotations are even less likely to provide a complete view.

With this in mind, recording the *provenance* of contributed annotations has been identified as a useful precaution to take in order that the origin of any given annotation can always be ascertained [19]. For now, this is managed organisationally, although several popular methods allow direct integration into the ontology. We are considering possible solutions to enable the competency question, ‘Under what conditions was this data collected?’ to be solved solely from the instance data and without making reference to organisationally held metadata.

### 5.6. Superfluity and Simplicity of Use

In conclusion to this section, we take a moment to consider the limitations of the SPHERE ADL ontology as it is presently expressed. The SPHERE ADL ontology is built using OBO tools and standards [37]. OBO is a relatively informal approach to the development of hierarchical concept representations, built in parallel to OWL [52] and intended for use within the biomedical sciences. We have already touched on one limitation—limited support for automated reasoning/validation. Consider the example of a potentially superfluous concept: the ontology explicitly defines both *alone* and *not alone*. As with most information structures of significant scale, the SPHERE ADL ontology contains a number of potentially superfluous concepts [53]. This specific example breaks the principle of orthogonality [54], since the two terms are closely coupled. One might reasonably ask why both are required, since negating the first implies the other.

In a pure OWL environment, it is possible to use automated reasoning to restrict and constrain values. For example, we might choose to formally express a concept such as ‘aloneness’ as being either true or false, so that our schema limits ambiguity in this area. OWL permits us to do so by formally describing *isAlone* as having a value of either ‘y’ or ‘n’, and nothing else—that is, a subject may either be alone or not alone, but cannot be both simultaneously. The ADL ontology does not presently permit automated validation to avoid such inconsistencies—such validation must occur *post hoc*. This approach, by contrast, means that widely used tools such as the reasoner made available in Protégé can be used to evaluate the validity of instance data, thus limiting our dependency on a hybrid toolchain that combines several tools.

OBO does not focus on automated reasoning, unlike OWL, and its ability to express relations between concepts is limited. However, it is much more convenient for use in the biomedical sciences due to the popularity of the language and tools. The practicality of direct implementation of automated validation using OWL or its more powerful cousin SWRL [55] is limited by the fact that many tools used in SPHERE do not directly support OWL or OBO, requiring instead a lossy transform into a simpler controlled vocabulary. As support for these standards continues to develop, an OBO-to-OWL mapping can be used on both ontology and instance data in order to enable their usage.

### 5.7. Ongoing Work Making Use of the SPHERE ADL Ontology

The future for the SPHERE ADL ontology involves new use cases with different and sometimes broader requirements. These may result in further evolution of the ontology in the future. Two examples of current and near future challenges are briefly presented here.

It is important to clarify that not every evaluation issue or unmet requirement means that the ontology must be revised. It is equally possible that another solution will resolve the issue. Potential mitigations for particular issues may include the decision to use an alternative knowledge structure for some or all of the requirements of a particular use case. For example, one might consider the possibility of combining elements from multiple ontologies or working from an ontology that can usefully be aligned with the SPHERE ADL ontology, rather than alteration of the ADL ontology to fully support an individual use case. In particular, as has been observed by Schraefel and Karger [56], it is important to recall that the ontology is an information artefact in a complex environment rather than a complete solution, and need not necessarily be displayed directly to the user; therefore, localisation [57], personalisation or any other customisation may be employed to improve outcomes without necessarily requiring change to the ontology itself. We have come to view this ontology as a concept spine of use for data description, particularly for data portability, support of reuse, data preservation and data management issues. It is possible that lightweight taxonomies or user-contributed labels undergoing subsequent analysis are more successful on a user-facing level: this ontology is not in general designed to be directly displayed to the user.

#### 5.7.1. DAta Post-Processing for Machine Learning Systems Development

In technology research, it is commonplace to make use of agile development methods when scheduling and specifying work, focusing on requirements currently expressed by stakeholders. The result is often to create products that are ‘sufficient unto the day’—able to support development of a solution to the immediate problem, but potentially inflexible and of limited broader application. However, in large studies, the costs, complexity and logistics of data collection and preprocessing are considerable. The process of annotation itself is a significant investment, irrespective of the complexity of the specific annotation schema selected for use. There are sound reasons to make use of a highly-structured approach to information annotation; although this increases costs relative to a simpler schema, it remains significantly cheaper than re-annotating a data set in toto.

Where supervised machine learning methods are used, classifier development for a given purpose generally requires a specific, concise ground truth tailored to that purpose. Given a set of annotations drawn from a well-structured, multi-tier ontology, it is straightforward to generate a simplified graph from a full timeline of annotations, fulfilling the functional requirements of the task. For example, an annotation set suitable for evaluating a location classification algorithm can be generated by filtering all annotations other than location from the graph. Similarly, selecting all annotations in the *hasPhysicalState* branch results in an annotation set suitable for exploring classifiers of physical motion—for example, developing a *sit to stand* transition classifier or timer.

#### 5.7.2. Preparing for Clinical Studies

SPHERE systems used as AAL sensor networks are expected to operate as a proxy for traditional instruments used in healthcare, such as clinical outcome measures, which are used to document and evaluate patient state and progress. With this in mind, clinicians were extensively involved in the initial development phases of the SPHERE ADL ontology. Data collection is now under way in the initial SPHERE studies, including the present ‘Hundred Homes’ study. In this deployment, a SPHERE home sensor network system is deployed in a wide variety of homes around Bristol. Several healthcare-focused studies associated with SPHERE have also begun, focusing on specific clinical conditions such as dementia and recovery from surgery such as hip and knee replacement.

The process of mapping relevant clinically related instruments (i.e., bases for measurement of patient condition) and outcome measures to the ADL ontology is now ongoing. To achieve this, it is useful to represent these clinical information structures as ontologies in their own right, mapping between them to establish correspondence or more complex relations or reasoning. These ongoing studies provide a useful opportunity to stress test the SPHERE ADL ontology, evaluating whether it is expressive enough to fully support the contexts in which it is used.

#### 5.7.3. Emerging Requirements

We expect ontology alignment, the process of mapping concepts between ontologies, to become increasingly significant for ubiquitous healthcare applications. This is in part a consequence of experiences such as recent work with healthcare professionals. We also expect that data provenance issues will increase in relevance as data reuse increases. The modality of data collection may have a significant effect on the quality and extent of data collected in tasks such as annotation [39] and it is likely that precise metadata regarding sensor and platform information will also come into play as the analysis of large deployment datasets continues. We propose that user interface-level schemas be stored alongside the parent ontology and any mapping information that is available, so that it is straightforward to identify, reuse and map between the knowledge representations used.

## 6. Discussion and Future Work

This article presents the SPHERE ontology of ADLs, two completed use cases and two ongoing use cases—machine learning and support for clinical studies. Ontology is a specification of a conceptualisation, i.e., it defines classes, their attributes and relationships between them in a particular domain of interest. Therefore it might be difficult to understand and use by users outside of the specific domain. AAL is one area of research which benefits vastly from the use of common, well-defined models. Such an approach eliminates any ambiguities and enables machines to reason over data; it also facilitates interdisciplinary, future-proof research. Ubiquitous systems are often linked to the Internet and contribute a large amount of data which is reasoned over not only by machines but also by stakeholders with different expertise.

The first use case presented in this article demonstrates how the SPHERE ontology of ADLs was used to annotate video ground truth data. Three tiers were assigned to describe activities in terms of level of detail, from high level activities to low level activities. Therefore, such ground truth is usable for validation of AR algorithms inferring activities at any of the three levels of granularity. Performing such video annotations required some in-depth knowledge of the ADL domain and hence annotators were briefed and trained to understand the modelled concept. Details of this study can be found in Woznowski et al. [42].

The second use case exposed the ontology to non-expert, untrained users facing the task of self-annotation of ADLs. Participants could log their activity via speech, NFC and through the use of buttons carrying activity labels, organised by room/location. This study is further described in Woznowski et al. [14] and Tonkin et al. [39]. Participants with no prior knowledge who faced this task had no problems understanding or using the provided tool. Hiding the complexity of the ontology in various software tools is very important. Otherwise, untrained, non-expert users may find such tools difficult to understand and impossible to use intuitively.

Beyond the internal uses of the SPHERE ontology presented in this article in the form of case studies, the ontology has been evaluated externally following its publication. We have made use of available publication and dissemination opportunities, participating in the series of recent ARDUOUS (Annotation of useR Data for UbiquitOUs Systems) workshops run in collaboration with the IEEE Conference on Pervasive Computing. As part of these workshops, this ontology has been benchmarked in use against alternative approaches, which has provided a valuable additional source of information about the strengths and weaknesses of each approach. This work is described in a recently released technical report [6]. Additionally, reuse and adaptation of the SPHERE ontology formed the basis for recent work at the University of Rostock, which approaches activity recognition using a rule-based modelling approach [58].

The SPHERE ontology of ADLs is to be used in clinical studies. SPHERE is a sensor platform for healthcare in residential environments and hence clinicians are interested in monitoring specific ADLs and behaviours. There is an ongoing need to map between the activities and states presented in this ontology and the conceptual graphs in use by clinicians—for example, an occupational therapist may evaluate a patient’s ability to complete variety of ADLs as outcome measures, including activities as straightforward as making a cup of tea [59].

Data preservation and publication together will represent the next big challenge for the SPHERE project. We expect that the use of the SPHERE ADL ontology will form part of our strategy for powering our data publication, indexing, selection and retrieval workflows, since the SPHERE dataset is significant in size and would be difficult to retrieve in full due to network and storage requirements. Consequentially, data indexing is a major focus of our attention. The ADL ontology is also expected to form part of our data preservation workflow, as the features identified within the dataset are part of commonplace data appraisal and selection workflows—although the content of a given dataset does not form part of the preservation metadata in itself, it is nonetheless part of various data preservation workflows. For example, data access, appraisal and retention decisions may be based on content metadata of this type.

We expect to continue working actively with domain experts to ensure the sustainability and relevance of the ontology beyond its initial publication, with the eventual aim of working towards standardisation within the community. This effort is not intended to exclude alternative approaches or standards, but to ensure that the user community for this ontology will continue to receive support beyond the lifetime of the original project and to enable active harmonization between standards in the future. Such active curation permits the outcomes of ongoing work, such as ontology alignment or internationalisation of labels, to be retained for future reuse. We hope that this will increase confidence in the SPHERE ADL ontology as a resource on which to draw for indexing and describing datasets, both for annotation purposes and for dataset publication and preservation.

Future work includes mapping unstructured text, such as that generated through voice input, to ontology terms. This involves extending this ontology with synonyms or linking to online dictionary services or linked data resources such as OntoWordNet. Interface design surrounding ontologies often falls prey to the so-called ‘pathetic fallacy of RDF’ [56]—the expectation that, because the underlying information structure has a certain form, in this instance a graph with concept labels, the resulting interface should display this directly. In practice, a concept synonym could be presented in the form of an utterance, a gesture, haptic interaction or an RFID activation, and the user may never view the ontology directly.

The authors plan to take this work further and make the result permanently available to the research community under an appropriate, extensible licence—something that many projects in this space, as demonstrated in the literature review, fail to do. To achieve this, further work is planned to include expression of the SPHERE ADL ontology in OWL, in order to overcome the currently identified limitations of the OBO toolset. This would provide support for automated reasoning/validation by, for example, defining stricter relationships between certain classes to eliminate superfluous concepts. Fully documenting, appropriately licensing, publishing and maintaining this ontology and its alignment with other standards of relevance to the domain would enable its widespread adoption and significantly increase data portability in this research domain.

## 7. Conclusions

Observation of the SPHERE ADL ontology in use has allowed us to identify strengths and weaknesses, as well as opportunities to develop the structure further. Work continues on alternative forms of data input, mapping and evaluation. For the purposes of automated reasoning and validation, we are exploring the possibility of making fuller use of existing mappings between OBO and OWL, opening up the potential for use of a wider variety of validation, constraint and mapping tools designed by the Semantic Web community. We expect to release further knowledge management resources, including revised materials built as a result of the work described here, supplementary materials created and used for specific use cases and further resources intended for purposes such as ontology alignment. Our work remains guided by practicality, alongside data quality metrics and concerns; we increasingly look to balance functional requirements against the requirements of data reusability, data management and data preservation, with long-term accessibility of the project outcomes and data as a key goal.

## Figures and Tables

**Table 1:** Activity classes in the SPHERE ADL ontology, including the highest and second highest ontology levels and the number of subclasses in each class.

Class	Subclasses	Example
Atomic home activities	7	
door interaction	3	open door
object interaction	6	pick up object
tap interaction	6	open hot tap
window interaction	2	close window
electrical appliance	4	switch on
cupboard interaction	2	open cupboard
draw interaction	2	open draw
Cleaning	17	mopping
Dishwashing	8	drying dishes
Eating/drinking	5	eating a meal
Exercising	6	stretching
Grooming	9	shaving
Health condition	6	coughing
Healthcare	3	treating a wound
Home env. management	9	water plants
adjusting light levels	2	switch light on
Hygiene	9	flossing
Information interaction	10	writing
using a computer	3	email
using a mobile phone/pda/...	4	sms
Laundry	11	ironing
Leisure	11	dancing
Meal/drink preparation	9	preparing a snack
Misc	1	smoking tobacco
Sleeping	5	napping
Social interaction	5	social media
talking	4	on a phone
Study-related	4	putting on sensors
Working	3	intellectual
Yardwork	3	gardening

**Table 2:** New and altered *activity* subclasses.

BoxLab	SPHERE ADL Ontology
Eating	Eating/drinking
Home management	Home environment management
Information	Information interaction
Meal preparation	Meal/drink preparation
—	Atomic home activities
—	Health condition
—	Misc
—	Social interaction
—	Working

**Table 3:** Physical state classes in the SPHERE ADL ontology.

Class	Subclasses	Example
Ambulation	9	crawling
Posture	7	kneeling
sitting	2	sitting on the floor
standing	2	standing still
Transitions	13	bending

**Table 4:** Room/location, social context and physiological context classes in the SPHERE ADL ontology.

Class	Subclasses	Example
Room/location	16	loft/attic
Social context	6	not alone
Physiological context	5	glucose level

**Table 5:** Desirable characteristics of an ontology in a given context of use.

Attribute	Source	Determination
Accuracy	[40]	Appropriate representation of aspects of the ‘real world’
Adaptability	[40]	Ease of performing changes
Consistency	[47]	Consistency of meaning of terms
Clarity/Interpretability	[40,47]	The extent to which defined terms/labels accurately convey the intended meaning
Cognitive adequacy	[40]	Match between formal and cognitive (user) semantic, effectively an indicator of cognitive load.
Conciseness	[40]	Absence of unnecessary definitions, axioms or complexity.
Completeness	[40]	Does the ontology cover all features required within the domain of interest?
Consistency	[40]	The extent to which consistent results are obtained from a given input (cf., inter-annotator consistency [48,49], inter-rater reliability [6])
Expressiveness	[40]	Does the ontology allow competency questions (or questions arising in use) to be answered?
Relevance	[47]	What proportion of the ontology maps to the context of use?
Precision/recall	[40]	Defined according to information science definitions: the relevance of returned information and the completeness of returned information.
Structural clarity	[50]	Formal specification/ontology must be free of cyclic references.

## Supplementary Materials

The SPHERE ontology of daily living is available online via the data.bris repository https://dx.doi.org/10.5523/bris.1234ym4ulx3r11i2z5b13g93n7.

## Abbreviations

The following abbreviations are used in this manuscript:

AAL	Ambient Assisted Living
ADL	Activities of Daily Living
AmI	Ambient Intelligence
AR	Activity Recognition
DCMI	Dublin Core Metadata Initiative
HCI	Human-Computer Interaction
ISO	International Organization for Standardization
NFC	Near Field Communication
OBO	Open Biomedical Ontologies
OWL	Web Ontology Language
RFID	Radio-frequency identification
SPHERE	Sensor Platform for HEalthcare in a Residential Environment
XML	Extensible Markup Language
W3C	World Wide Web Consortium
